# Resveratrol-Induced Vascular Progenitor Differentiation towards Endothelial Lineage via MiR-21/Akt/β-Catenin Is Protective in Vessel Graft Models

**DOI:** 10.1371/journal.pone.0125122

**Published:** 2015-05-11

**Authors:** Paola Campagnolo, Xuechong Hong, Elisabetta di Bernardini, Ioannis Smyrnias, Yanhua Hu, Qingbo Xu

**Affiliations:** Cardiovascular Division, King’s College London BHF Centre, London, United Kingdom; William Harvey Research Institute, Barts and The London School of Medicine and Dentistry, Queen Mary University of London, UNITED KINGDOM

## Abstract

**Background and Purpose:**

Vessel graft failure is typically associated with arteriosclerosis, in which endothelial dysfunction/damage is a key event. Resveratrol has been shown to possess cardioprotective capacity and to reduce atherosclerosis. We aimed to study the influence of resveratrol on the behavior of resident stem cells that may contribute to graft arteriosclerosis.

**Experimental Approach:**

Vascular resident progenitor cells and embryonic stem cells were treated with resveratrol under differentiating conditions and endothelial markers expression was evaluated. Expression of miR-21 and β-catenin was also tested and exogenously modified. Effects of resveratrol treatment in an ex vivo re-endothelialization model and on mice undergone vascular graft were evaluated.

**Key Results:**

Resveratrol induced expression of endothelial markers such as CD31, VE-cadherin and eNOS in both progenitor and stem cells. We demonstrated that resveratrol significantly reduced miR-21 expression, which in turn reduced Akt phosphorylation. This signal cascade diminished the amount of nuclear β-catenin, inducing endothelial marker expression and increasing tube-like formation by progenitor cells. Both the inhibition of miR-21 and the knockdown of β-catenin were able to recapitulate the effect of resveratrol application. Ex vivo, progenitor cells treated with resveratrol produced better endothelialization of the decellularized vessel. Finally, in a mouse model of vessel graft, a resveratrol-enhanced diet was able to reduce lesion formation.

**Conclusions and Implications:**

We provide the first evidence that oral administration of resveratrol can reduce neointimal formation in a model of vascular graft and elucidated the underpinning miR-21/Akt/β-catenin dependent mechanism. These findings may support the beneficial effect of resveratrol supplementation for graft failure prevention.

## Introduction

Resveratrol (trans-3,4,5’-trihydroxystilbene) is a natural phytochemical also available as a dietary supplement, originally derived from grapes and naturally occurring in red wine and some Asian medicinal herbs. Recent findings have reported a role for resveratrol in the so-called ‘French paradox’, the epidemiological observation of the relatively low incidence of cardiovascular diseases in the high saturated fat-consuming French population. In this context, resveratrol has been shown to induce an anti-inflammatory phenotype in endothelial cells [1], protecting them from apoptosis and reducing platelet and macrophages adhesion/extravasation [2]. Furthermore, low doses of resveratrol have been shown to enhance reendothelialization and to reduce neointima formation after endothelial injury by acting on endothelial nitric oxide synthase (eNOS) expression and activity and endothelial progenitor cells homing [1,3]. To date, no study has reported the effect of resveratrol on the differentiation of vascular resident stem/progenitor cells.

Our group has previously isolated and characterized a population of vascular progenitor cells that participate to the repopulation of the decellularized scaffold, used in our mouse model of vessel graft. These cells express progenitor markers, such as Sca-1 and CD90 and are able to give rise to both endothelial and smooth muscle cells *in vitro* [4]. Interestingly, recent reports demonstrated that vessel wall progenitor cells contribute to neointimal formation in vein grafts [5–7]. Typically, the insurgence of neointimal growth after vessel graft is initiated by the loss of the endothelial layer, due to extensive cell death, which triggers inflammatory response and vascular smooth muscle cell proliferation [8]. Studies from our group and others have also demonstrated that damaged cells in vessel grafts can be replaced by blood and vessel-derived progenitor cells [7,9]. In this process, the changes in micro-environmental clues (i.e. VEGF) can modulate the differentiation of the resident progenitor cells and induce endothelial differentiation, therefore reducing neointimal formation [4]. In this study, we propose a novel mechanism of action for resveratrol in the context of graft atherosclerosis. Indeed, we established that resveratrol is able to influence stem cell and resident progenitor cell fate, inducing endothelial differentiation and therefore reducing neointimal formation. Furthermore, we analyzed the downstream pathway leading to endothelial differentiation and established that resveratrol acts through the inhibition of the miR21/Akt/β-catenin pathway.

## Material and Methods

Detailed Material and Methods can be found in the online-only Supplement.

### Cell culture

Mouse embryonic stem cells ES cell (ES-D3 cell line, CRL-1934; ATCC, Manassas, VA) were cultured as previously reported [10]. Sca-1+ vascular resident progenitor cells were obtained as previously described by spontaneous migration from decellularized vessel grafts collected 2 weeks after implantation [4]. Differentiation was induced by plating cells on Collagen IV-coated flasks in presence of differentiation medium (DM) containing alpha DMEM (Gibco) supplemented with 10% FBS (Gibco), 0.2mM 2-mercaptoethanol and 100u/ml penicillin and 100μg/ml streptomycin for 3 days, followed by 5 days of treatment with or without the addition of 20μM resveratrol.

### Decellularized vessel seeding

Decellularized vessel was prepared as previously described by isolating a mouse thoracic aorta and treating it with SDS [4]. The vessel obtained was then seeded with 1×10^6^ ESC differentiated for 3 days in DM with or without the addition of 20μM resveratrol. Vessels were harvested after 6 days of resveratrol or normal medium circulation.

### Animal procedure and lesion measurement

Animal experiments were performed in accordance with UK and European legislation under the Animals (Scientific Procedures) Act 1986 and European Directive 2010/63/EU and approved by King’s College London Institutional Animal Care and Use Committee. ApoE-/- mice were fed with normal chow or RSV chow (0.02% in weight, calculated intake 24mg/kg/day) for 7 days before venous graft was performed. Vein graft procedure was similar to that described previously [11]. Animals were fed with resveratrol or control diet for additional two weeks and lesion area was measured as described before [12].

### Statistical analysis

Data are presented as mean±SEM and are representative of at least three independent experiments. Student’s t-test was used to compare 2 samples, one-way ANOVA for multiple comparisons followed by pair-wise comparison.

## Results

### Resveratrol induces endothelial marker expression in stem/progenitor cells

**Fig 1:**
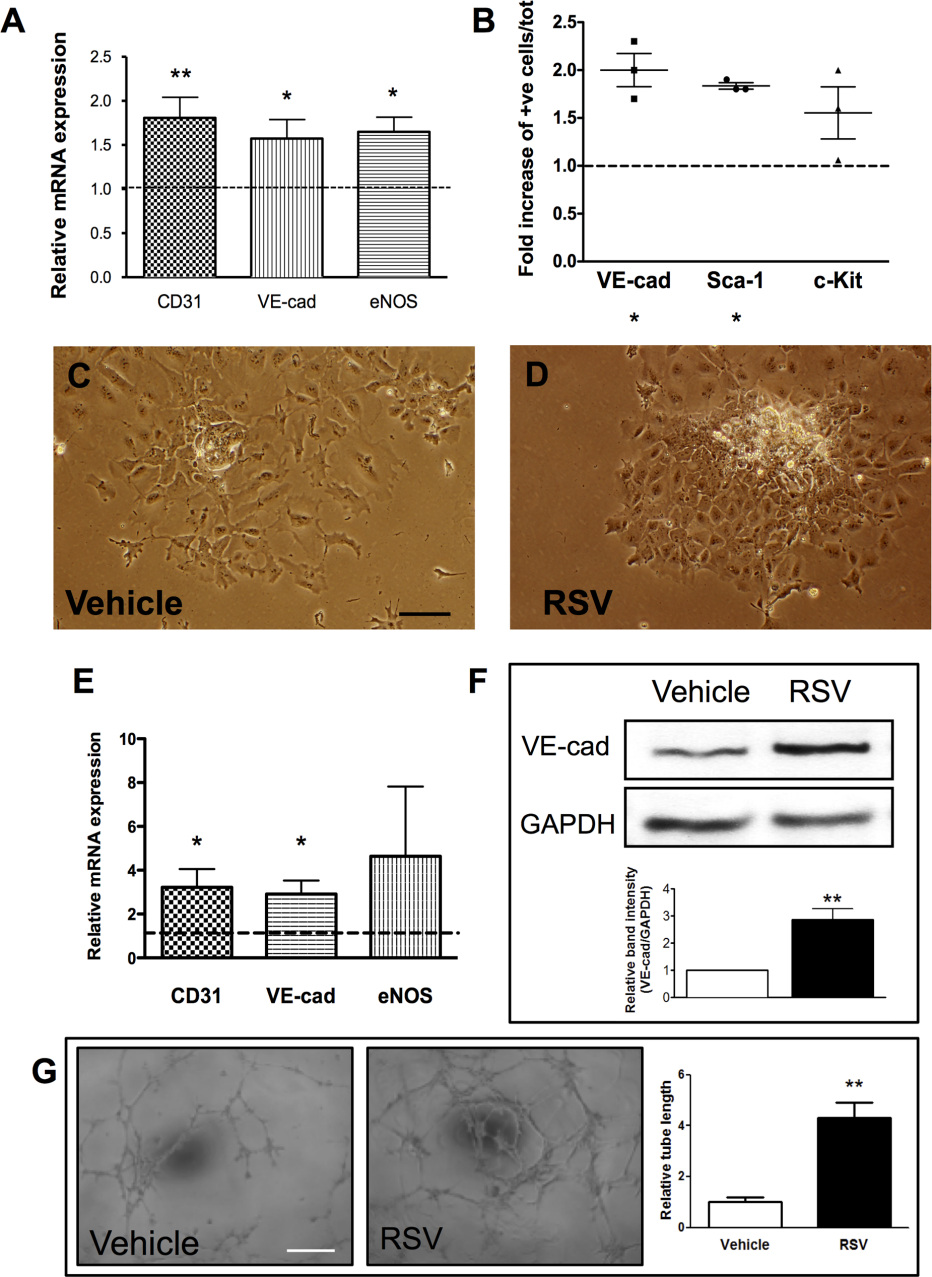
Effect of resveratrol on stem and progenitor cell marker expression. Embryonic stem cells (ESC, A-D) and vessel-derived progenitor cells (E-H) were differentiated in the presence of 20μM resveratrol (RSV) for 5 days. In ESC, gene expression analysis showed increase in endothelial marker expression CD31, VE-cadherin and eNOS, as compared to control medium (A). Flow cytometry analysis showed increased percentage of cells expressing VE-cadherin and progenitor markers Sca-1 and c-Kit (B). Microphotographs showed the different morphology of cells cultured in differentiation medium without (C) and with resveratrol (D). In vessel-derived progenitor cells treatment with resveratrol increased the gene expression of CD31 and VE-cadherin and eNOS (E) as well as the protein levels of VE-cadherin (F), as compared to control. Tube formation capacity was increased by resveratrol treatment, as compared to control (G). * p<0.05 and **p<0.01. Scale bars indicate 100μm.

We tested the ability of resveratrol to induce endothelial marker expression in pre-differentiated embryonic stem cells. ESCs were plated on collagen IV for 3 days and then treated with resveratrol for further 5 days. Gene expression analysis showed that addition of resveratrol to the culture medium induced the expression of endothelial markers such as CD31, VE-cadherin and eNOS as compared to the control medium-treated cells (Fig 1A). Flow cytometry analysis confirmed the increase in the percentage of cells positive for VE-cadherin and also showed an increase in the number of cells expressing vascular progenitor markers, such as Sca-1 and c-Kit (Fig 1B). Accordingly, we observed that the cells cultured in normal differentiation medium assumed an elongated morphology typical of smooth muscle cells, while in presence of resveratrol they formed small colonies with cobblestone appearance, resembling endothelial cells (Fig 1C and 1D).

Next, we examined the effect of resveratrol on the differentiation of the resident progenitor cell population previously described by our group to be involved in the repopulation of a decellularized vessel graft *in vivo*. These cells have been shown to express progenitor markers and to be able to differentiate to both endothelial and vascular smooth muscle cells and to contribute to neointima formation in a mouse model of graft [4]. We hypothesized that the differentiation of these cells might influence the progression of the lesion. As previously described, graft-derived resident progenitor cells expressed Sca-1 and mesenchymal markers, when cultured in non-differentiating conditions (S1 Fig). Treatment with resveratrol induced the expression of endothelial markers in the progenitor cells, as shown at both gene expression and protein levels (Fig 1E and 1F). Furthermore, resveratrol increased the ability of the progenitor cells to form tube-like structure in an *in vitro* angiogenesis assay, as compared to control medium (Fig 1G). Analysis of smooth muscle marker expression showed no difference at mRNA and promoter activity level (S2 Fig).

In order to extrapolate the contribution of proliferation and cell death on the differentiative effect of resveratrol, we cultured the graft-derived progenitor cells with or without resveratrol and proceeded to measure cell number (proliferation), apoptosis (CaspaseGlo 3/7 activity). We tested concentrations ranging from 5 to 100μM, including the concentration of 20μM that was used in all the differentiation experiments. Results showed that cell number was unaffected between 5 to 20μM. Reduction in cell number was observed in presence of 100μM of resveratrol (S3 Fig). Concomitantly, the activity of Caspase 3/7 was dramatically increased presence of 100μM of resveratrol, indicating cytotoxicity but remained unaffected in all other conditions (S3 Fig).

Taken together, these results showed that 20μM resveratrol is able to induce markers of endothelial differentiation in both stem and progenitor cells without substantial affecting their proliferation and apoptosis.

### Resveratrol reduces miR-21 expression regulating endothelial marker expression

**Fig 2:**
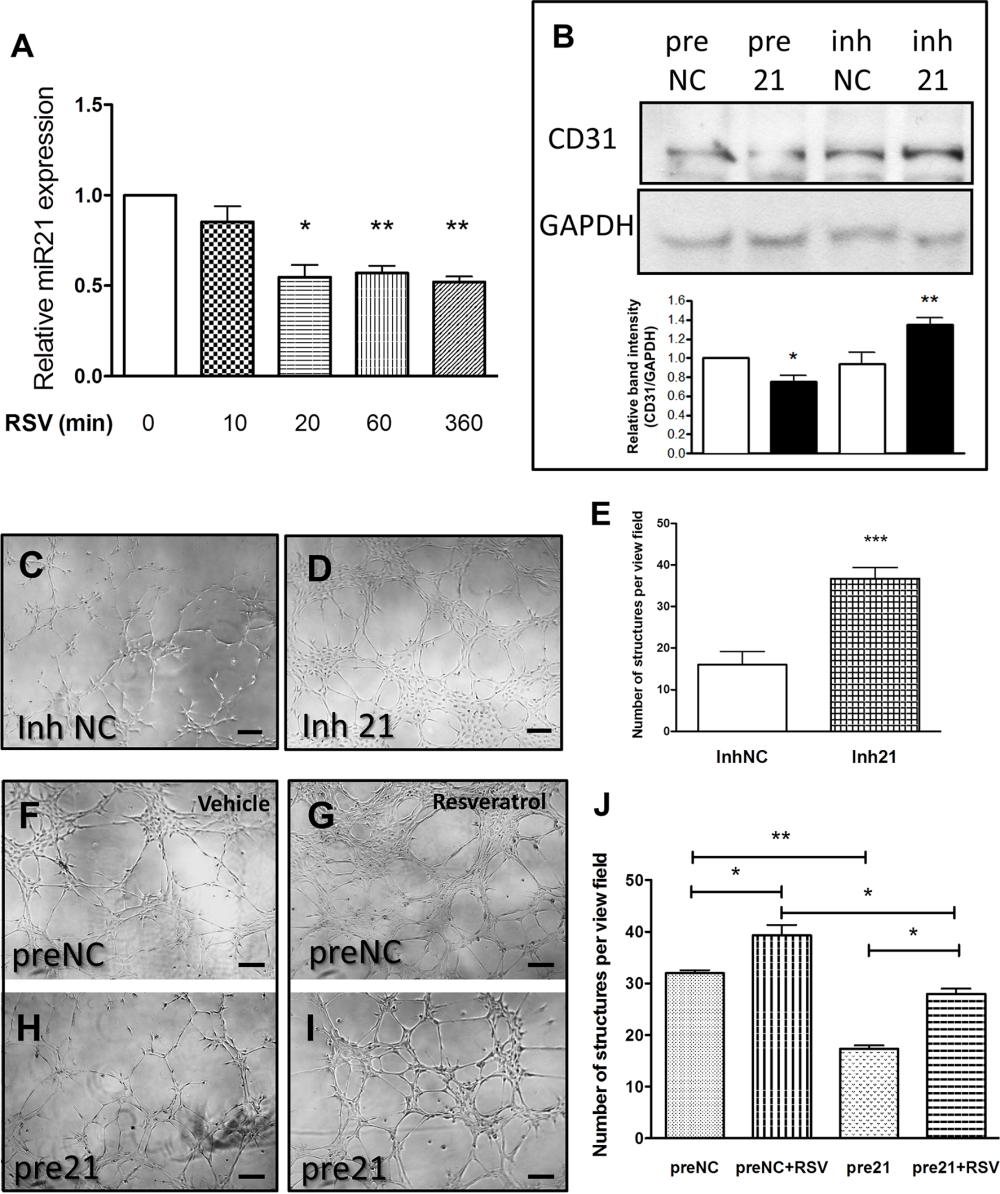
Inhibition of miR21 by resveratrol regulates induces endothelial marker expression. Treatment of the progenitor cells with resveratrol (RSV) inhibited miR21 expression (A). Overexpression of miR21 (pre21) decreased CD31 protein level, as compared to the control transfected cells (preNC) (B). MiR21 downregulation (inh21) increased CD31 expression, as compared to the negative control (inhNC) (B). Tube-formation was increased by Inh21 (D), as compared to control treated cells (C), as confirmed by total tube length quantification (E). Representative pictures (F-I) and total tube length quantification (J) showed that the resveratrol (RSV)-dependent increase in tube length was counteracted by miR-21 transfection. *p<0.05, **p<0.01 and ***p<0.001. Scale bars indicate 50μm.

Resveratrol has been previously shown to regulate the expression of miR-21 in the context of cancer cell growth and metastasis [13,14]. On the other hand, miR-21 is known to exert a role in angiogenesis, although its effect seems to be context dependent [15,16]. We decided to study the role of miR-21 in the resveratrol-dependent differentiation of the vascular resident progenitor cells. Firstly, we studied the time course of miRNA-21 expression in cells treated with resveratrol and established that the expression level of miR-21 was reduced after resveratrol treatment for up to 6 hrs (Fig 2A). In order to establish whether the modification of miR-21 expression plays a role in the differentiation, we overexpressed miR-21 using a precursor (pre-21) or downregulated it using an inhibitor (inh-21). Transfected cells were cultured for 4 days in collagen IV-coated plates to induce differentiation and endothelial marker expression and tube formation capacity were assessed. Results showed that overexpression of miR-21 reduced the levels of CD31 protein, while the inh-21 treatment had the opposite effect (Fig 2B). Interestingly, inhibition of miR-21 increased the ability of progenitor cells to form tube-like structures *in vitro*, as compared to the control (Fig 2C–2E). Furthermore, we verified the tube-like forming capacity of the progenitors after application of resveratrol with or without miR-21 overexpression. *In vitro* angiogenesis assay showed that in cells treated with the control pre-miR, resveratrol increased the tube formation capacity (Fig 2F and 2G). The forced expression of miR-21 sensibly reduced the tube-formation capacity of the progenitor cells, both in presence or absence of resveratrol (Fig 2H and 2I). Total tube length quantification confirmed the observation that miR-21 reduces tube formation capacity of progenitor cells and blunts resveratrol-dependent increase in structure formation (Fig 2J).

**Fig 3:**
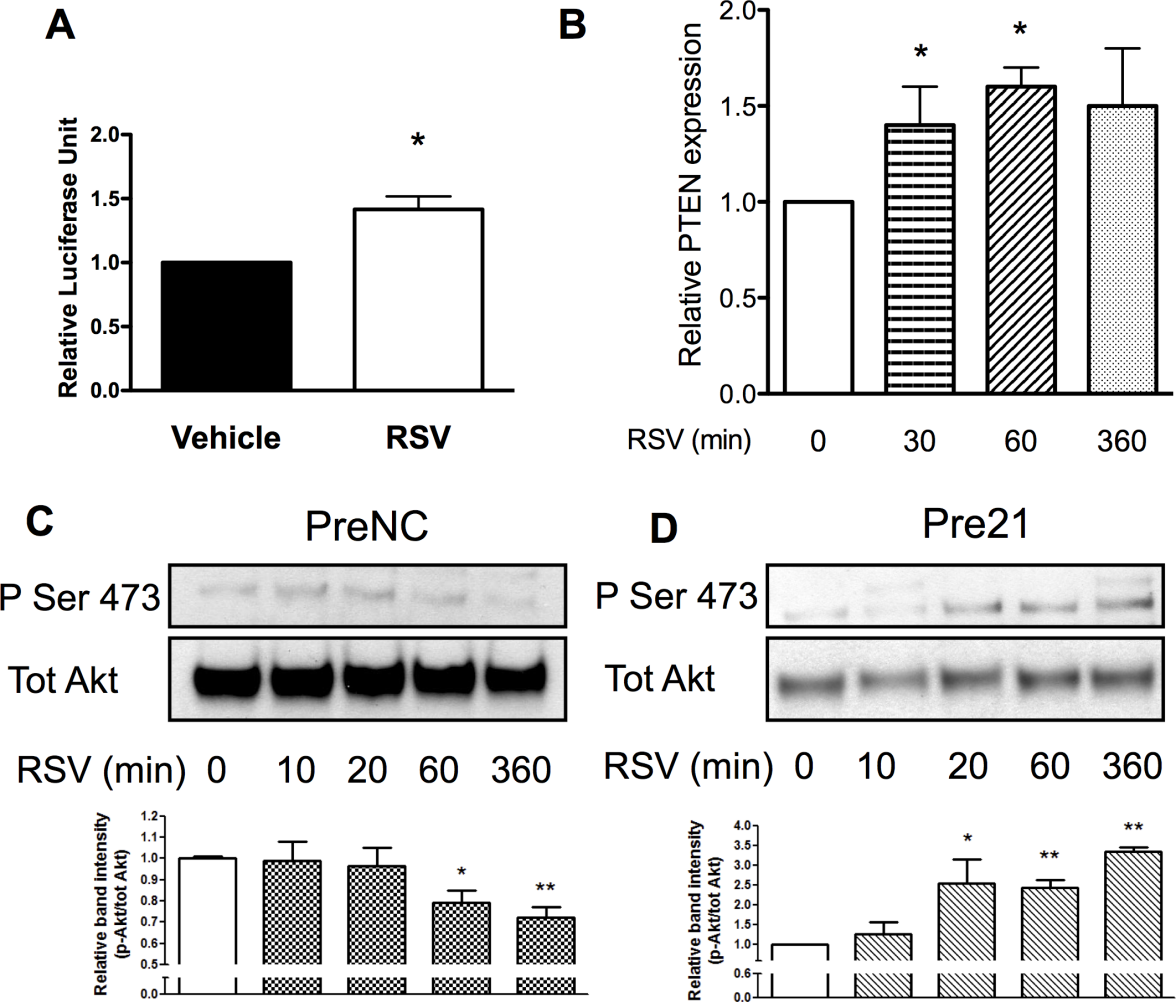
Resveratrol inhibits Akt pathway through miR21. Luciferase assay of the PTEN 3’UTR showed increased activity when vessel derived progenitor cells were treated with resveratrol (RSV) for 48h, as compared to control (Vehicle, A). Gene expression analysis confirmed increased expression of PTEN (B). Protein analysis indicated a reduction of phosphorylation of Akt after resveratrol treatment (C), this effect was reversed by miR21 overexpression (D), *p<0.05.

Previous studies have demonstrated that miR-21 directly binds and induces the degradation of phosphatase and tensin homolog (PTEN) mRNA, a well-known inhibitor of Akt phosphorylation [16]. We therefore analyzed the effect of resveratrol and miR-21 treatments on the PTEN/Akt pathway. Cells were transfected with a plasmid bearing the luciferase reporter gene under the control of the PTEN 3’UTR, which contains the miR-21 binding site. Following the transfection, cells were treated with resveratrol and luciferase activity measured. Analysis showed an increase in luciferase activity after resveratrol treatment, indicating a reduction in miR-21 binding to the promoter (Fig 3A). Consistently, gene expression levels of PTEN were increased shortly after resveratrol stimulation (Fig 3B). Furthermore, we assessed the levels of phosphorylation of Akt and established that treatment with resveratrol reduces Akt phosphorylation after 60 min and this effect is maintained for 6hrs (Fig 3C). In presence of mi-R21 overexpression, this pattern of phosphorylation was reverted and phosphorylation of Akt resulted to be increase after 1 and 6 hours (Fig 3D). Therefore, these data demonstrated that miR-21 expression and its downstream target PTEN/Akt pathway are altered by resveratrol treatment and are involved in the expression pattern of progenitor cells.

### Effect of resveratrol on endothelial markers expression is mediated by the inhibition of β-Catenin

**Fig 4:**
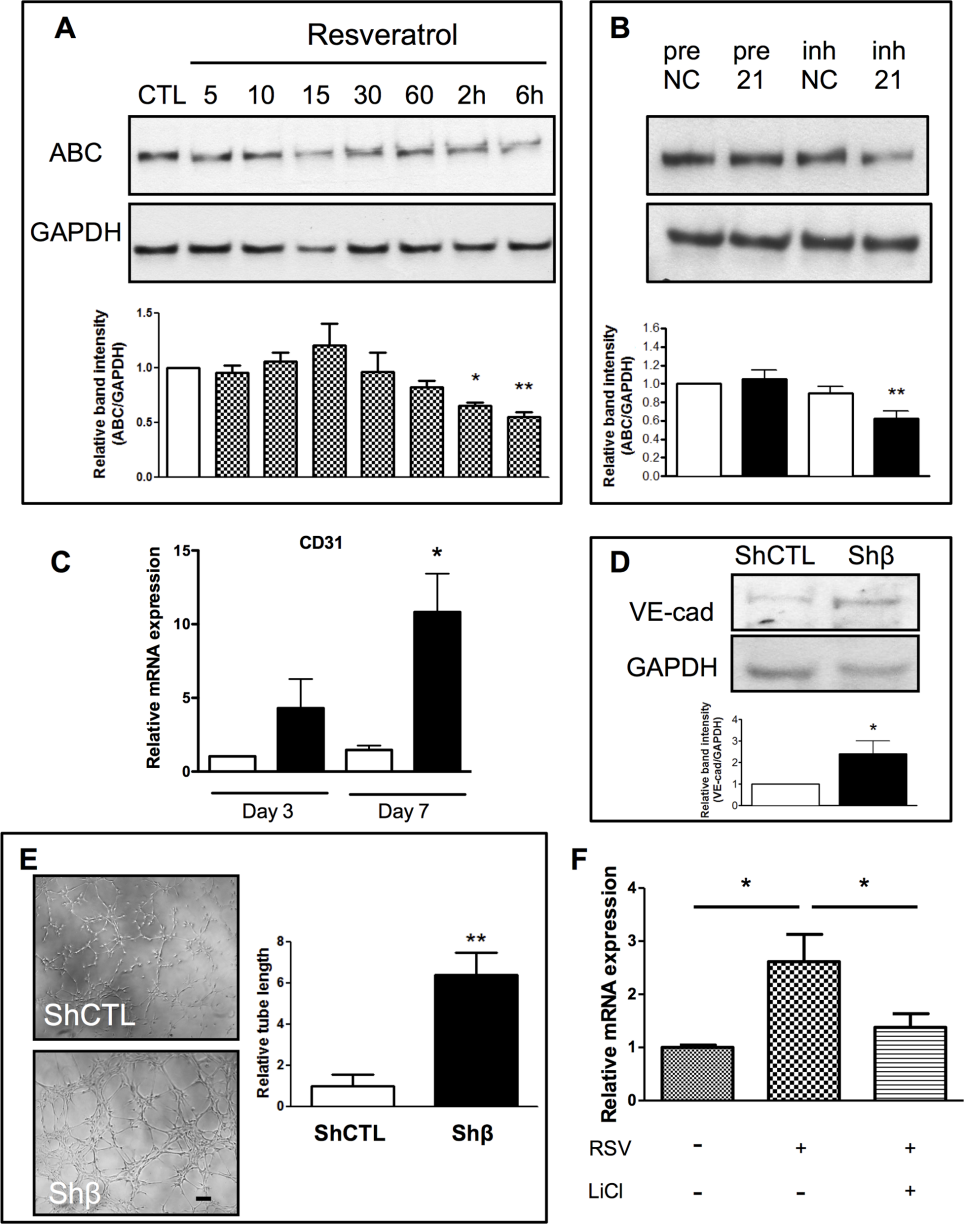
Inhibition of β-catenin by resveratrol and inh21 enhances endothelial marker expression. Levels of activated β-catenin (ABC) were reduced by resveratrol treatment (A). Transfection of vessel resident progenitor cells with miR21 inhibitor (inh21) decrease ABC expression, as compared to the negative control (inhNC, B). No changes were associated with miR21 overexpression (pre21, B). Silencing of β-catenin by lentiviral infection (black bars) induced the expression the endothelial marker CD31 (C), as compared to control virus (empty bars). β-catenin inhibition (Shβ) increased VE-cadherin protein level, as compared to the control virus (ShCTL, D). Representative images showing the increase in tube-formation capacity of Shβ-catenin infected cells, as compared to control cells (ShCTL, E). β-catenin activation by LiCl (50mM) was able to counteract resveratrol (RSV)-induced CD31 overexpression (F). *p<0.05 and **p<0.01. Data are representative of three independent experiments. Scale bar indicates 50 μm.

Previous studies have shown that β-catenin is directly regulated by the Akt pathway [17] and act as a mediator of resveratrol in the induction of chondrogenic differentiation of mesenchymal stem cells [18]. We hypothesized the contribution of β-catenin in the context of endothelial differentiation of vessel resident progenitor cells. Western blot analysis showed that resveratrol treatment reduced the levels of active β-catenin in resident progenitor cells (Fig 4A). Additionally, we observed that cells treated with the inhibitor of miR-21 showed reduced levels of active β-catenin (Fig 4B). To study the effect of β-catenin in the differentiation process, we employed lentiviral-mediated silencing that allowed for long term knockdown of β-catenin (S4 Fig). Measurement of the expression of genes associated with differentiation at 3 and 7 days showed an increase in CD31 expression after β-catenin silencing (Fig 4C) and an increase in VE-cadherin protein expression (Fig 4D). Moreover, progenitor cells lacking β-catenin formed more tube-like structures on Matrigel, as compared to the control-infected cells (Fig 4E). In order to confirm the contribution of β-catenin in the resveratrol-dependent EC marker expression, we treated the progenitor cells with resveratrol and stimulated the canonical Wnt pathway by adding LiCl. As shown in Fig 4F, stimulation of β-catenin pathway by LiCl blunted the effect of resveratrol on CD31 expression. Taken together, these results show that resveratrol and miR-21 modify β-catenin activation, subsequently affecting progenitor cell markers expression.

### Resveratrol promotes endothelialization ex-vivo and reduces neointima formation in a mouse model of vascular grafts

**Fig 5:**
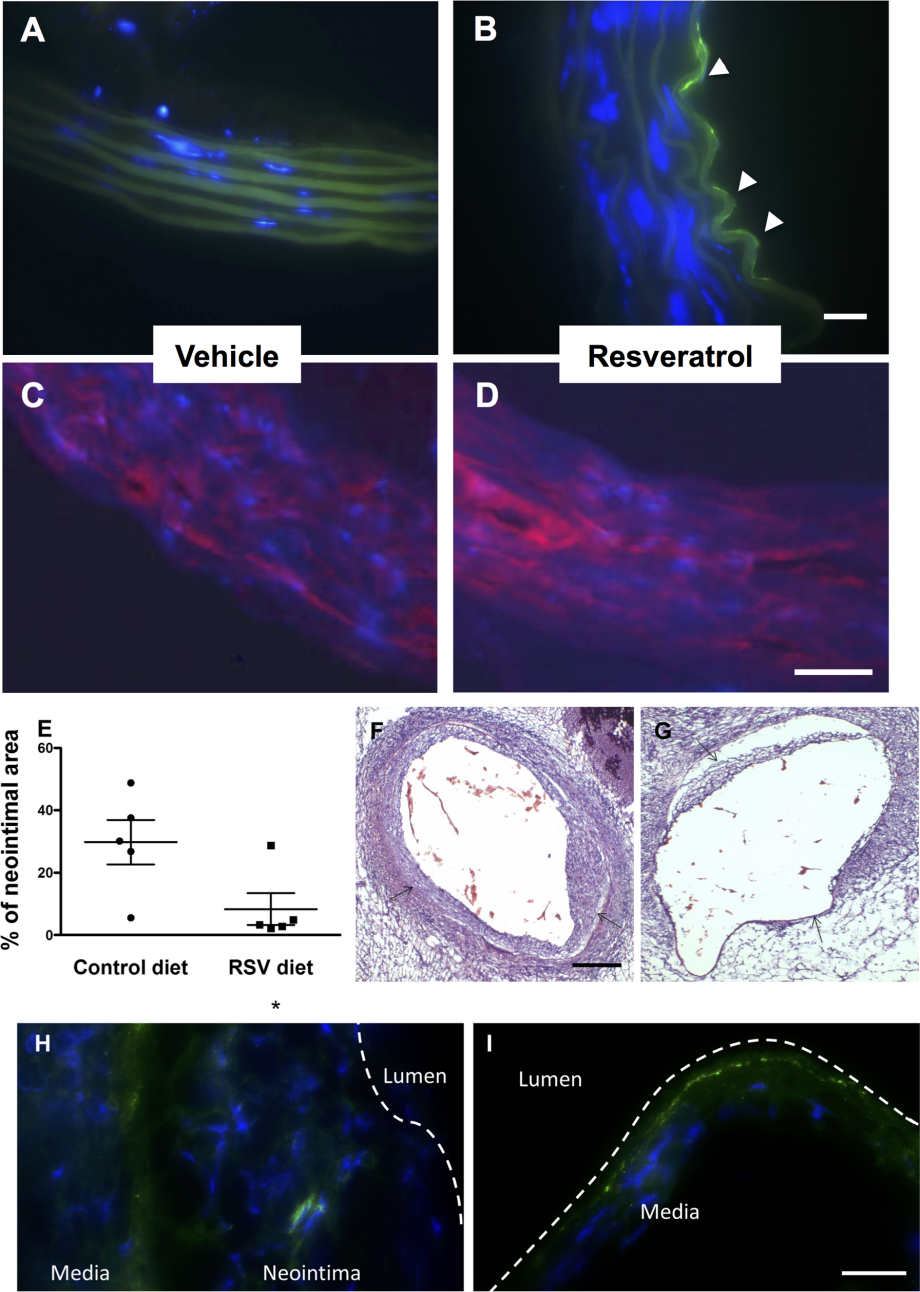
Effect of resveratrol on *ex vivo* and *in vivo* re-endothelialization and neointimal formation. Embryonic stem cells were pre-differentiated for 3 days in the presence or absence of resveratrol and then seeded on a decellularized vessel in an ex-vivo bioreactor. The resveratrol treated cells were fed with resveratrol-containing circulating medium, while the control cells were fed normal medium. Representative images showing the lack of endothelial coverage on decellularized vessels infused ex vivo with stem cells differentiated in presence of control medium (A) as compared to the extensive re-endothelialization observed with resveratrol-treated cells (B, CD31, white arrowheads). No difference was observed in SMA staining (C and D). ApoE-/- mice were fed normal chow or resveratrol-chow for 7 days before undergoing vascular graft (n = 5 per group). Diet as continued for further 14 days and then graft sections were stained and analyzed. Animal fed with resveratrol-chow showed reduced neointimal area within the graft, as compared to normal diet-fed animals (E). Representative H&E images showed thicker neointimal layer in control (F, black arrow) as compared to resveratrol-treated animals (G). Immunostaining for CD31 on the sections showed less endothelial staining from normal diet-fed animals (H). Animals fed with resveratrol-chow showed an intact endothelial layer (I, CD31). DAPI, blue: nuclei. Scale bars: 25μm (A-D and H and I) and 200μm (F and G).

Our group has devised an *ex-vivo* model of vascular graft in which a decellularized vessel scaffold can be repopulated by progenitor cells, using a bioreactor connected to a pump, providing the medium flow [19]. We used this *ex-vivo* system to study the effect of resveratrol on in the ability of stem cells to differentiate into endothelial cells and repopulate the intimal layer of the decellularized vessel. Pre-differentiated embryonic stem cells were treated for 3 days with resveratrol or control medium and then placed in the circulation of the bioreactor in the presence of resveratrol or control medium for additional 6 days. Vessels harvested were stained for the endothelial marker CD31, showing the development of a continuous luminal endothelial layer specifically in the vessel treated with resveratrol (Fig 5A and 5B). Staining for smooth muscle marker SMA showed uniform distribution in the media of the repopulated vessels, independently from the treatment (Fig 5C and 5D).

To further verify our hypothesis, we studied the effect of resveratrol in an *in vivo* model of arterial graft. The vena cava was grafted into the carotid artery of ApoE-/- mice, which were fed normal chow or resveratrol containing diet, starting 7 days before the operation. Samples were collected after 15 additional days of different diet and the formation of neointimal lesion was quantified on sections obtained from formalin fixed grafts. Data showed that animals fed with diet supplemented with resveratrol presented significantly reduced neointimal lesions, as compared to mice fed with a normal diet (Fig 5C–5E). Furthermore, staining for CD31 showed improved endothelial coverage in the resveratrol-fed animals (Fig 5F and 5G). Thus, we demonstrated that resveratrol increased endothelialization in *ex-vivo* settings and reduced neointimal formation *in vivo* after vessel graft, potentially through the increase of endothelial differentiation and coverage.

## Discussion

Epidemiological studies have demonstrated a sensible discrepancy in the incidence of cardiovascular events in populations consuming a moderate amount of red wine [20,21]. This phenomenon is referred as the ‘French paradox’ and has been partially attributed to the presence of resveratrol in the red wine. While some of the mechanisms implicated in the effect of resveratrol in the context of cardiovascular diseases have been elucidated, no studies have reported its effect on vessel resident progenitor cell differentiation. Resveratrol has been shown to reduce thrombogenesis and atherogenesis in fat-fed hypercholesterolemic mice [22,23] and to improve the outcome of aortic aneurysm by reducing inflammation and leukocyte infiltration [24]. Furthermore, resveratrol was able to reduce neointimal formation after endothelial injury through its protective effect on endothelial cell integrity [1,2]. In line with previously published findings, in the present study we established that oral administration of resveratrol was able to reduce the formation of neointimal lesions in the context of vascular graft model in ApoE-/- mice. These results have an important clinical relevance for the use of resveratrol as a supplement to improve vascular graft patency.

It was found that reduction of neointimal growth was associated with better endothelial coverage and intact intimal layer. Following this observation, we propose a novel atheroprotective mechanism for resveratrol, through the induction of endothelial differentiation of vessel resident progenitor cells. The promotion of a prompt protective endothelialization after grafting is important for the positive long-term outcome of vessel graft, since it limits thrombogenesis and stenosis. We studied the effect of resveratrol on Sca-1+ progenitor cells that were previously isolated and characterized by our group. Sca-1+ progenitors were shown to participate to graft repopulation and to be prompted by local stimuli to differentiate into both vascular smooth muscle and endothelial cells [4]. Indeed, we established that treatment of vascular resident progenitor cells with resveratrol increased their endothelial marker expression; these results were confirmed using mouse embryonic stem cells. The role of resveratrol in angiogenesis has been mainly studied in the context of tumorigenesis, where it was shown to reduce capillary formation by inhibiting the release of pro-angiogenic factors by cancer cells [25,26]. Interestingly, recent papers have elucidated an important role of resveratrol in increasing the number, viability and function of bone marrow endothelial progenitor cells [3,27,28]. It is of note that we did not observe an overall effect of resveratrol on the vessel-derived progenitor cells proliferation, as was previously observed for blood derived endothelial progenitor cells [28–30]. This discrepancy might reflect the different nature of the two cell types, with bone marrow endothelial progenitor cells committed almost exclusively to the endothelial lineage and therefore proliferating to produce more endothelial cells, while the mesenchymal-like vessel-derived progenitors are activated towards the endothelial differentiation pathway. Our results shed a new light on the role of resveratrol on endothelial cell differentiation and function by demonstrating its influence on vascular resident progenitor cells.

MiRNAs are short non-coding nucleotides that have recently been shown to regulate the expression of a large number of genes and have been therefore implicated in numerous physiological and pathological processes, including differentiation, angiogenesis and atherosclerosis. One of the most important observations in the present study is the finding of a novel mechanism underlying the resveratrol-dependent induction of endothelial marker expression. In our system, treatment with resveratrol strongly reduced miR-21 expression, leading to increased PTEN expression and reduced Akt phosphorylation. We also demonstrated that miR-21 overexpression inhibited endothelial differentiation via the increase of Akt phosphorylation. Interestingly, this pathway is the reverse of the TGFβ-induced endothelial-to-mesenchymal transition, where TGFβ induces miR-21 and activates the Akt pathway, decreasing endothelial marker expression [31]. This is quite remarkable considering the mesenchymal nature of the described resident progenitor population. Moreover, these results are in accordance with previous studies on cancer cells showing inverse correlation between resveratrol and miR-21 [13,14], while miR-21 has been shown to reduce angiogenesis in mature endothelial cells [15]. More importantly, the aberrant overexpression of miR-21 has been shown to cause increase neointimal formation in a rat model of balloon injury via PTEN inhibition [32]. In addition, the results recently published by McDonald et al on the beneficial effect of miR-21 knockout in a model of vessel graft complement and support our findings on the role of resveratrol in the same model and confirm the central role played by miR-21 in this mechanism [33].

**Fig 6:**
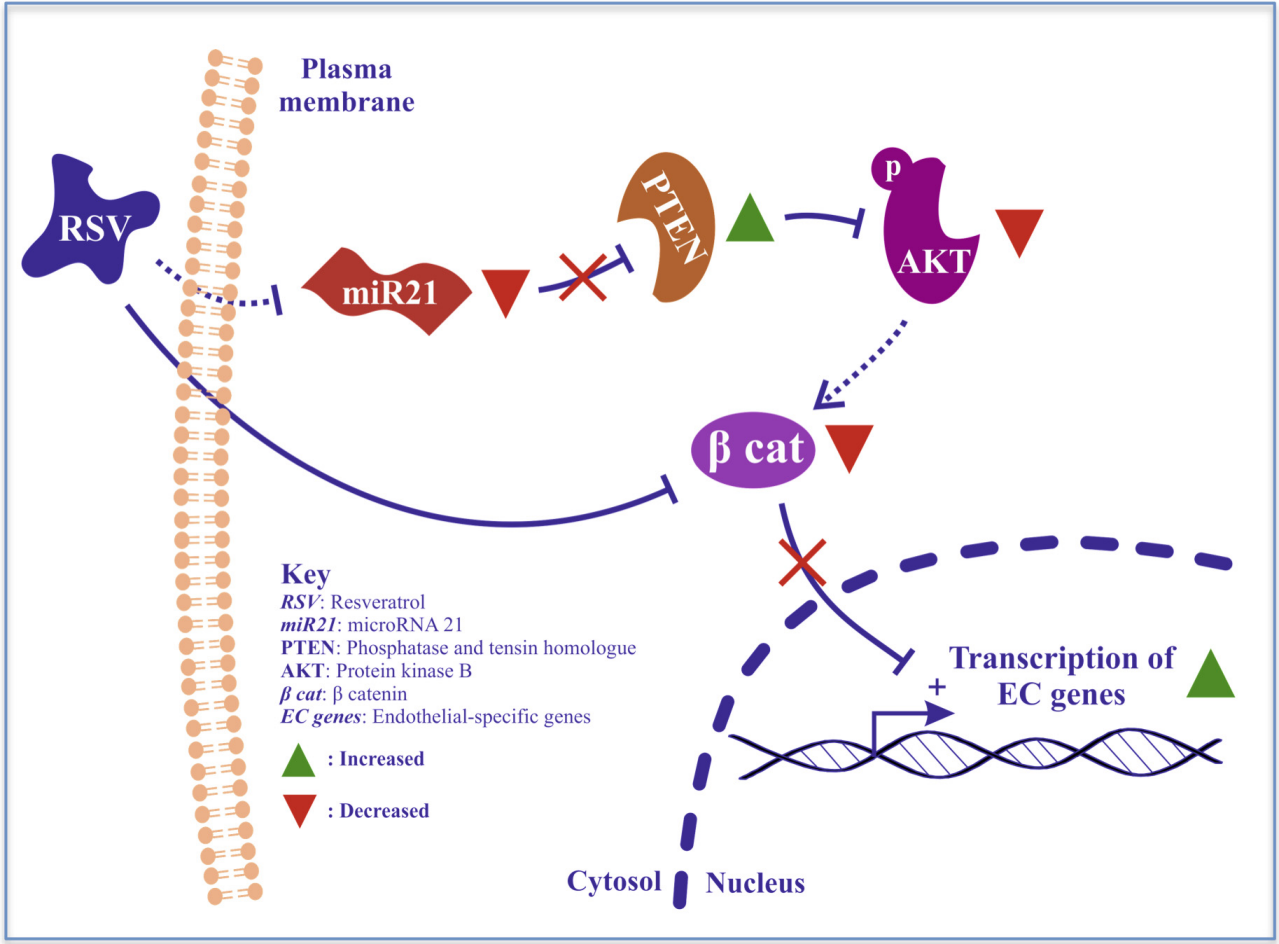
Schematic representation of the mechanism proposed. In the system, resveratrol is shown to reduce miR-21 expression and leads to the increased expression of phosphatase and tensin homolog (PTEN), Akt inhibitor. PTEN overexpression decreased Akt phosphorylation. In parallel, β-catenin active form was reduced, either due to the reduction in Akt activity or through an independent pathway. Reduction of β-catenin and miR-21 inhibition both affect the status of vascular resident progenitor cells, inducing their endothelial marker expression.

Previous work has demonstrated that Akt can directly activate β-catenin; the reduced activation of Akt observed after resveratrol treatment can therefore be at least partially responsible for the detected diminished activation of β-catenin [34]. Inhibition of β-catenin via lentiviral-mediated knockdown increased endothelial marker expression and tube-formation capacity in the progenitor cell population, indicating a contribution of the β-catenin suppression to their functional differentiation. In the present study, we have established that both the application of resveratrol and the downregulation of miR-21 decreased Akt and β-catenin activation, thus promoting differentiation in the progenitor cells. This finding is consistent with the previously published papers showing that similar doses of resveratrol negatively regulate the activation of β-catenin, reducing angiogenesis in mature endothelial cells and inhibiting cancer cell growth [18,35]. The discrepancy of effects of resveratrol in different cell types can be explained by the context-dependent function of β-catenin. Thus, the implication of β-catenin in the pathway creates additional evidence of the link between resveratrol and miR-21 by providing a common effector (Fig 6).

In summary, we established that resveratrol exert its role in directing stem/progenitor cell differentiation towards endothelial lineages by inhibiting miR-21 and therefore reducing Akt activation and β-catenin translocation. Resveratrol can also promote cell repopulation of a decellularized vessel *ex vivo* and reduce neointimal formation in an *in vivo* model of vascular graft. Therefore, the mechanisms elucidated in this work not only provides further insight in the pharmacological action of resveratrol, but might also suggest new clinical applications in vascular diseases where endothelial differentiation/coverage is affected.

## Supporting Information

S1 Fig Characterization of vascular resident progenitor cells. Representative images showing the homogeneous expression of progenitor/mesenchymal markers CD34 (A), CD44 (B), Sca1 (C) and CD90 (D).
(TIFF)

S2 Fig Effect of resveratrol on smooth muscle marker expression. Vessel derived progenitor cells were cultured in presence of resveratrol (RSV) for 5 days. Real time PCR showed no changes in smooth muscle marker expression (A) and luciferase assay indicated no significant difference in the activity of SMA promoter (B).
(TIFF)

S3 Fig Effect of resveratrol treatment on progenitor cells growth and cells death. Progenitor cells were treated with differentiation medium containing 0 to 100μM of resveratrol and tested for proliferation (Alamar Blue, A) and apoptosis (CaspaseGlo 3/7, B). ***p<0.001.
(TIFF)

S4 Fig Quantification of silencing efficiency. Representative real time PCR results showing the effective knockdown of β-catenin (A) and the consequent downregulation of the downstream gene Axin2 (B) at day 3 and 7 after infection with silencing lentivirus (Sh beta catenin, black columns) as compared to control (Sh control, white column).
(TIFF)

S1 File Supporting Materials and Methods. Additional materials and methods.
(PDF)

S1 Table Supporting table. Primer sequences.
(PDF)
