# Supplementary figures and images for: Resveratrol-Induced Vascular Progenitor Differentiation towards Endothelial Lineage via MiR-21/Akt/β-Catenin Is Protective in Vessel Graft Models
Source: PLoS One. 2015 May 11;10(5):e0125122. doi: 10.1371/journal.pone.0125122 (PMC4427364; doi:10.1371/journal.pone.0125122)

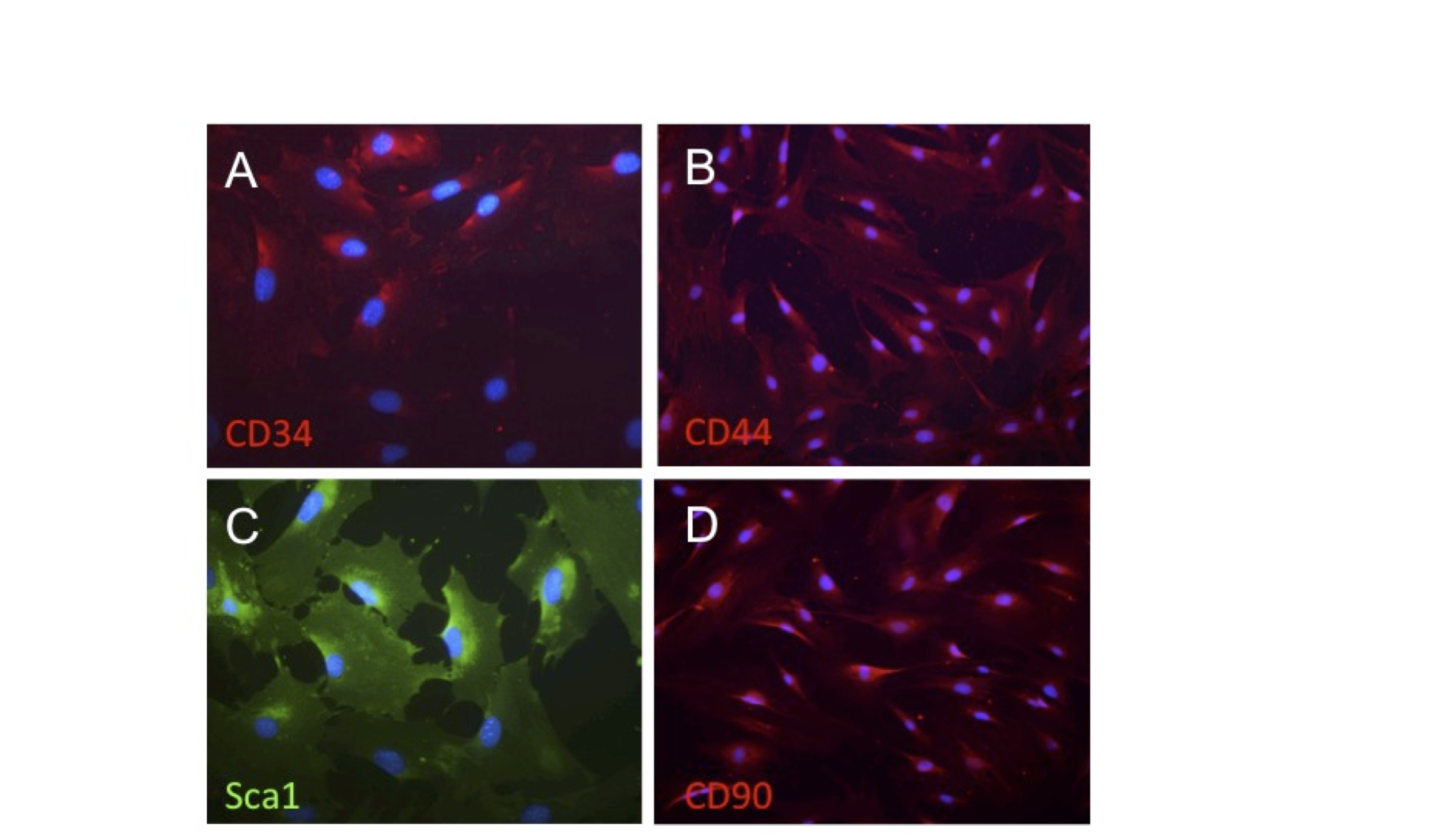

Supplement: S1 Fig — Representative images showing the homogeneous expression of progenitor/mesenchymal markers CD34 (A), CD44 (B), Sca1 (C) and CD90 (D). (TIFF) [file pone.0125122.s001.tiff]

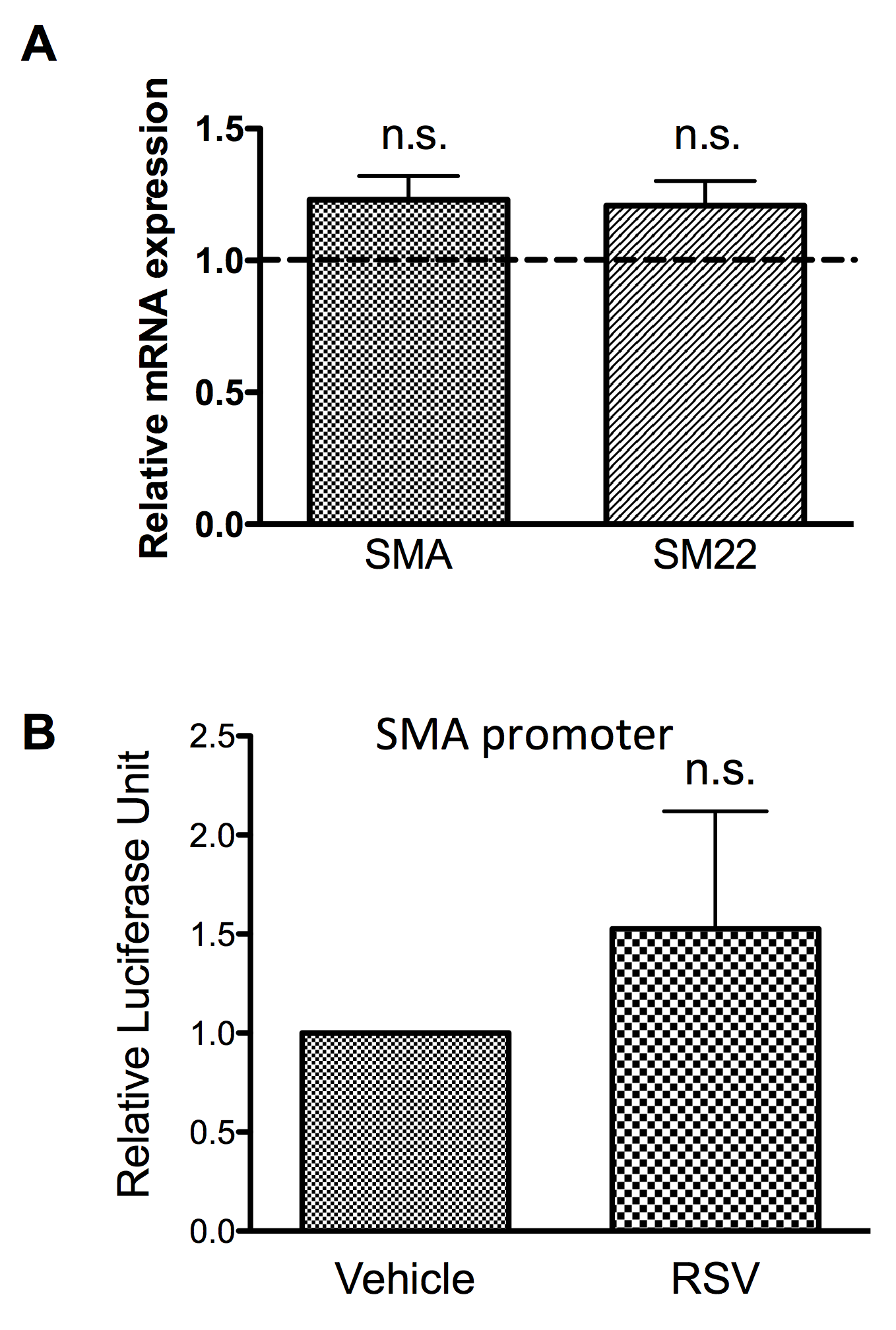

Supplement: S2 Fig — Vessel derived progenitor cells were cultured in presence of resveratrol (RSV) for 5 days. Real time PCR showed no changes in smooth muscle marker expression (A) and luciferase assay indicated no significant difference in the activity of SMA promoter (B). (TIFF) [file pone.0125122.s002.tiff]

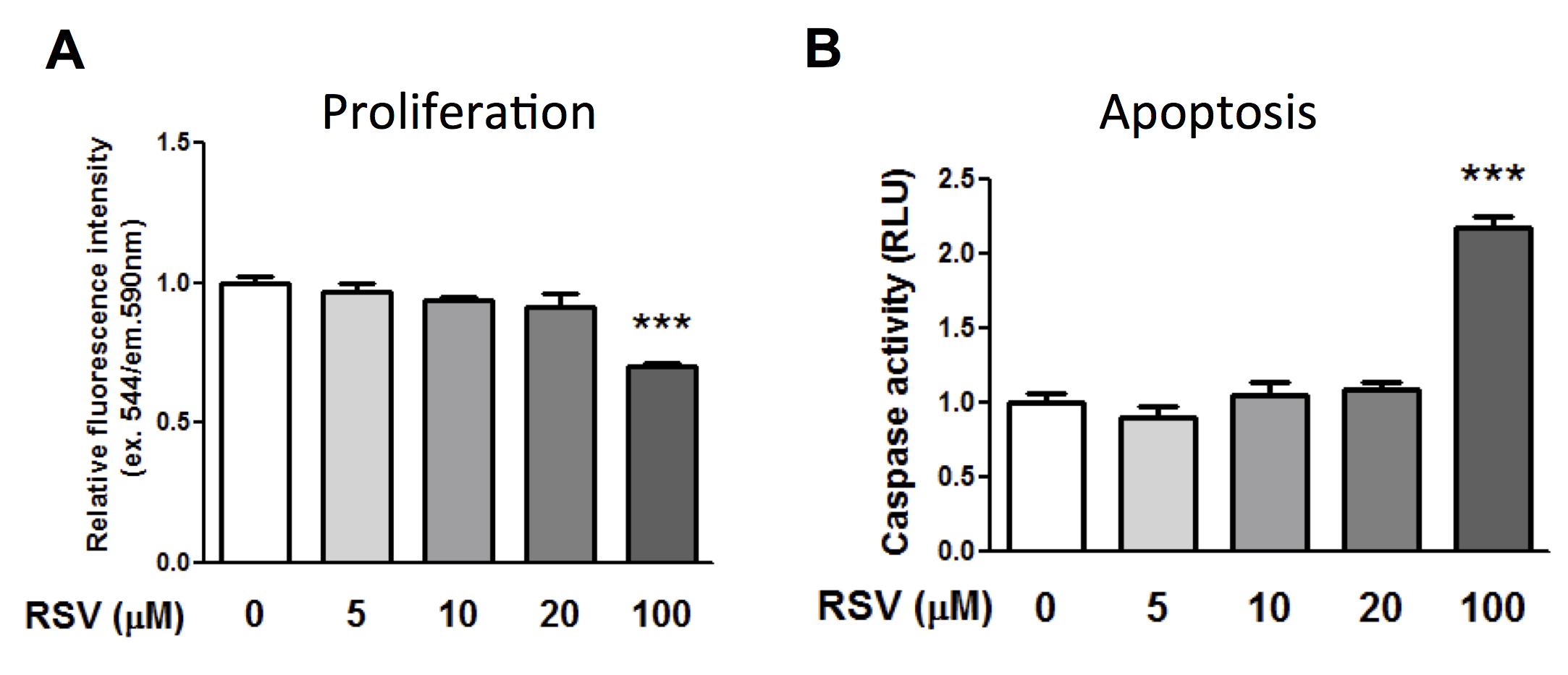

Supplement: S3 Fig — Progenitor cells were treated with differentiation medium containing 0 to 100μM of resveratrol and tested for proliferation (Alamar Blue, A) and apoptosis (CaspaseGlo 3/7, B). ***p<0.001. (TIFF) [file pone.0125122.s003.tiff]

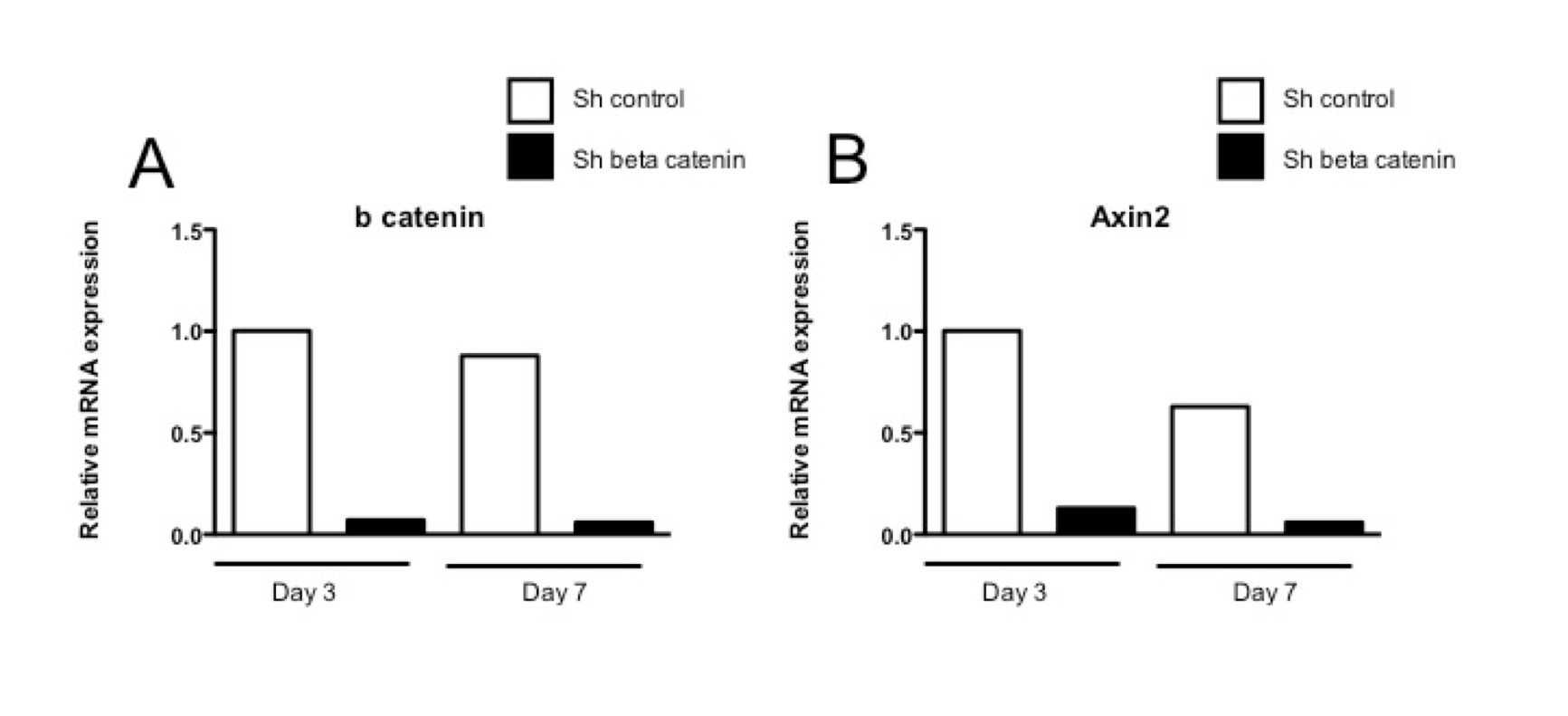

Supplement: S4 Fig — Representative real time PCR results showing the effective knockdown of β-catenin (A) and the consequent downregulation of the downstream gene Axin2 (B) at day 3 and 7 after infection with silencing lentivirus (Sh beta catenin, black columns) as compared to control (Sh control, white column). (TIFF) [file pone.0125122.s004.tiff]
