# Supplementary material for: Resveratrol-Induced Vascular Progenitor Differentiation towards Endothelial Lineage via MiR-21/Akt/β-Catenin Is Protective in Vessel Graft Models
Source: PLoS One. 2015 May 11;10(5):e0125122. doi: 10.1371/journal.pone.0125122 (PMC4427364; doi:10.1371/journal.pone.0125122)
Supplement: S1 File — Additional materials and methods. (PDF) [file pone.0125122.s005.pdf]

Supporting Material and Methods

Materials

Antibodies were purchased from the following sources: goat anti-VE-cadherin (SC-6458) and goat anti-CD31 (sc-1506) antibodies were purchased from Santa Cruz Biotech (Santa Cruz, CA, USA). Mouse anti-GAPDH, rabbit anti-VE-cadherin (ab33168), mouse CD90 (ab225), rat CD34 (ab8158) and rabbit IgG isotype controls were purchased from Abcam. FITC-conjugated anti-Sca-1 (553335) was purchased from BD Pharmigen. PE-conjugated anti-VE-cadherin (12-1441-80) and anti-cKit were purchased from ABBiotech and BD, respectively. Mouse anti-activated β-catenin (ABC) was purchased from Millipore (05-665). The secondary antibodies were purchased from Invitrogen. Recombinant human VEGF was purchased from Peprotech. Resveratrol was purchased from Sigma and used at a concentration of 20μM (R5010). LiCl was purchased from Sigma and used at a concentration of 50mM.

Cell culture, isolation and differentiation

Mouse embryonic stem cells (ES-D3 cell line, CRL-1934; American Type Culture Collection [ATCC]) were cultured as previously reported.[1] Briefly, cells were cultured in 0.04% gelatin-coated flasks in DMEM (ATCC) supplemented with 10% FBS (EmbryoMax, Millipore), 10ng/ml LIF (Millipore), 0.1mM 2-mercaptoethanol, 100 U/ml penicillin, and 100 μg/ml streptomycin in a humidified incubator supplemented with 5% CO_2, and they were split at a 1:6 ratio every other day. Vascular resident progenitor cells were isolated as previously described by harvesting

decellularized vessel graft after 2 weeks from implantation and letting the cells migrate out of the explants.[2] Sca-1+ progenitors were isolated using MACS magnetic beads (Miltenyi) and cultured in stem cell medium containing LIF. Differentiation was induced by plating cells on collagen IV-coated flasks in presence of differentiation medium containing alpha DMEM (Gibco) supplemented with 10% FBS (Gibco), 0.2mM 2-mercaptoethanol and 100u/ml penicillin and 100µg/ml streptomycin.

RNA and miRNA extraction, reverse transcription and real time PCR

Total RNA was extracted from cell pellets following the instructions of RNeasy kit (Qiagen, Crawley, UK) and reverse transcribed (MMLV, Promega). Quantitative gene expression analysis was performed using Eppendorf Realplex2 Mastercycler and mix in combination with the primers listed in the Supporting Information Table 1.

Extraction of total RNA including miRNA was performed using miRNeasy Mini Kit (Qiagen), according to the manufacturer's protocol. 20ng of RNA from each sample was reverse transcribed using the Megaplex RT Primers (Rodent Pools A) and pre-amplified using Megaplex PreAmp Primers and Mastermix from Applied Biosystem, before relative expression of miR-21 and controls was evaluated using Taqman miRNA Assay.

In vitro tube formation assay

Functional differentiation of progenitor cells was assessed as previously described.[3] Briefly, cells were differentiated for 5 days in presence of a specific stimulus and a

total of 3×10^4 cells were placed in each well of an 8-well chamber slide (BD) on top of 100µl of Matrigel (BD). Tube-like structure formation was monitored at 4-6h. Images were acquired with Axioplan 2 imaging and were processed with ImageJ.

Immunoblotting and immunofluorescence staining and flow cytometry

Western blot analysis was performed on cell pellets lysed in RIPA buffer; 20-50µg of proteins from each sample was loaded on 6-8% polyacrylamide gels and standard immunoblotting procedure was performed. Immunostaining was performed as described before[4] on adherent cells fixed in 4% buffered paraformaldehyde and blocked in 5% BSA before incubation with primary antibody for VE-cadherin. For flow cytometry, cells were detached and resuspended in staining medium (PBS containing 1% FBS) and stained with directly conjugated Sca-1, VE-cadherin, c-Kit antibodies and analysed using FACSCalibur, BD.

Cell proliferation and apoptosis measurement

Progenitor cells were cultured as described above, cells were detached and 1500 cell/ well were plated on black walls/clear bottom plates coated with Collagen IV. The following day differentiation medium containing DMSO or resveratrol at 5, 10, 20 or 100µM was added, cells were tested after 2 days. Alamar blue (Life technologies) was added 1:10 to the culture medium and cells were incubated at 37 degrees and 5% CO_2 for 3h, before fluorescence (ex.544, em.590nm) was read using Spectramax M5 plate reader (Molecular Devices).

Apoptosis was measured using CaspaseGlo 3/7 (Promega) following the manufacturer protocol. Briefly, CaspaseGlo reagent was added to the medium in 1:1 ratio and the

reaction was incubated in the dark for 1h. Luminescence originated from Caspase 3/7 activity was measured using Spectramax M5 plate reader (Molecular Devices). Measurements were normalized by the average value of the control (Vehicle).

Transient transfection and lentiviral infection

Luciferase assay was performed by transfecting cells with FuGene, according to manufacturer's instruction. Plasmid carrying luciferase reporter gene under the control of the 3'UTR-PTEN was obtained from Addgene (pGL3-control-PTEN-3'UTR-WT plasmid 21326, Joshua Mendell laboratory)[5] and co-transfected into the differentiating cells together with a control plasmid encoding for Renilla. 6h after transfection cells were treated with 20μM resveratrol. Renilla and luciferase activity were detected 48hr later using a standard protocol. Relative luciferase unit (RLU) was defined as the ratio of luciferase activity to Renilla activity with that of control set as 1.0. SMA promoter Luciferase plasmid was prepared as reported in [6].

MiRNA expression levels were modified by transiently transfecting the cells at 60–70% confluence with pre-21, the negative control precursor miRNA (Ambion), miR-21 inhibitor and the negative control of miRNA inhibitor (Exiqon) using Lipofectamine™ RNAiMAX (Invitrogen) in serum-free alpha MEM medium, according to the manufacturer's instruction. The final concentration of the oligomers was 50μM for inh-21 and the inh-NC and 5μM for pre-21 and the pre-NC. After 5 hours, an equal volume of fresh medium was added to the transfection medium.

Lentivirus-mediated silencing was obtained using short hairpin RNA plasmids obtained from SigmaMission database, specific for β-catenin or non-coding sequence.

The infection was performed in the presence of 10μg/ml of polybrene (Millipore) for 18h.

Decellularized vessel preparation and seeding

The preparation of the decellularized vessels was performed as previously described.[2] Briefly, the thoracic aorta was excised from mouse and immediately flushed with saline solution to prevent blood clot formation. The vessel was treated in 0.075% sodium dodecyl sulphate (SDS) solution (Severn Biotech Ltd, UK) overnight and then washed in PBS. The vessel was fixed in the incubation chamber of a special customized circulation bioreactor (Zyoxel Ltd, Oxford, UK). The complete setup was maintained in a standard CO2 incubator at 37°C. ESCs were pre-differentiated for 3 days on collagen IV-coated plates with or without resveratrol and then 1×10^6 cells were seeded in each decellularized vessel via direct injection. After 12 hours incubation to allow the attachment of the cells to the vessel, shear stress generated from medium flow (with or without resveratrol) was applied. The medium flow was driven by a peristaltic pump (Masterflex L/S Digital Drive, model 7520; Standard Pump head, model 7018-20, Codane Tubing, Cole-Parmer, UK). Shear stress was applied at stepwise rates ranging from 5 to 20 dynes/cm^2 over a period of 36 hours. No increase in the shear stress rate was conducted after 48 hours and the vessels were harvested on day 6. The circulating medium was changed every other day.

Mouse model of vein grafts

All animal experiments were performed according to protocols approved by the Institutional Committee for Use and Care of Laboratory Animals. ApoE-deficient mice were purchased from Charles River (Margate, Kent, UK). The mice were randomly divided in two groups, one group was fed with normal chow, the second group was fed with chow containing 0.02% of resveratrol (with a calculated uptake of resveratrol of 24mg/kg/day, as previously described[7]) for 7 days before venous graft was performed.

The procedure used for vein graft was similar to that described previously.[8] Briefly, 3-month-old mice were anesthetized using a combination of Hypnorm (25 mg/kg body weight; Veta Pharma, UK) and Hypnovel (25 mg/kg, Roche) administered introperitoneally. Atropine sulphate (1.7 mg/kg) was given together in the combination to prevent the obstruction of respiratory tract by salivary secretion. Each mouse was fixed in a supine position with its neck extended. The right common carotid artery was mobilized free from the bifurcation at the distal end toward the proximal end at thoracic inlet. The middle portion of common carotid artery was ligated twice with an 8-0 silk suture and dissected between two ties, and cuffs (0.65 mm in diameter and 1 mm in length) made of autoclavable nylon tube (Portex, London, UK) were placed over the ends. The artery was inverted over the cuffs and ligated with 8-0 silk sutures. The vena cava conduit harvested from an isogenic donor mouse was grafted as an interposition graft by sleeving the vein over the two ends of the carotid artery and ligating them with 8-0 silk sutures. Vigorous pulsations in the grafted vessel confirmed successful engraftment. Animals were fed with resveratrol-enhanced chow or control diet for additional two weeks after the procedure. Grafts were harvested after two weeks and used for downstream analysis.

Lesion Measurement

Frozen sections were obtained from different points of the harvested grafts and stained with hematoxylin and eosin and lesion area was evaluated as described before[9] by subtracting the area of the lumen from the area enclosed by the line inside of the media. Additionally, 5 µm-thick frozen sections were fixed in a cold acetone 10 minutes, stained with primary antibody against CD31 (1:100; BioLegend) and counterstained with DAPI.

Statistical analysis

Data is expressed as the mean ± the SEM was analyzed with a two-tailed t test, for two groups, or one-way ANOVA followed by pair-wise comparisons. A value of $p < 0.05$ was considered to be significant.

1. Vittet D, Prandini MH, Berthier R, Schweitzer A, Martin-Sisteron H, Uzan G, Dejana E. Embryonic stem cells differentiate in vitro to endothelial cells through successive maturation steps. *Blood*. 1996; 88:3424–3431.

2. Tsai T-N, Kirton JP, Campagnolo P, Zhang L, Xiao Q, Zhang Z, Wang W, Hu Y, Xu Q. Contribution of Stem Cells to Neointimal Formation of Decellularized Vessel Grafts in a Novel Mouse Model. *Am. J. Pathol.* 2012; 181:362–373.

3. Campagnolo P, Cesselli D, Haj Zen Al A, Beltrami AP, Krankel N, Katare R, Angelini G, Emanueli C, Madeddu P. Human adult vena saphena contains perivascular progenitor cells endowed with clonogenic and proangiogenic potential. *Circulation*. 2010; 121:1735–1745.

4. Zeng L, Xiao Q, Margariti A, Zhang Z, Zampetaki A, Patel S, Capogrossi MC, Hu Y, Xu Q. HDAC3 is crucial in shear- and VEGF-induced stem cell

differentiation toward endothelial cells. *The Journal of Cell Biology*. 2006; 174:1059–1069.

5. O'Donnell KA, Wentzel EA, Zeller KI, Dang CV, Mendell JT. c-Myc-regulated microRNAs modulate E2F1 expression. *Nature*. 2005; 435:839–843.

6. Margariti A, Zampetaki A, Xiao Q, Zhou B, Karamariti E, Martin D, Yin X, Mayr M, Li H, Zhang Z, De Falco E, Hu Y, Cockerill G, Xu Q, Zeng L. Histone Deacetylase 7 Controls Endothelial Cell Growth Through Modulation of beta-Catenin. *Circulation Research*. 2010; 106:1202–1211.

7. Delucchi F, Berni R, Frati C, Cavalli S, Graiani G, Sala R, Chaponnier C, Gabbiani G, Calani L, Del Rio D, Bocchi L, Lagrasta C, Quaini F, Stilli D. Resveratrol treatment reduces cardiac progenitor cell dysfunction and prevents morpho-functional ventricular remodeling in type-1 diabetic rats. *PLoS ONE*. 2012; 7:e39836.

8. Zou Y, Dietrich H, Hu Y, Metzler B, Wick G, Xu Q. Mouse model of venous bypass graft arteriosclerosis. *Am. J. Pathol.* 1998; 153:1301–1310.

9. Zeng L, Zampetaki A, Margariti A, Pepe AE, Alam S, Martin D, Xiao Q, Wang W, Jin Z-G, Cockerill G, Mori K, Li Y-SJ, Hu Y, Chien S, Xu Q. Sustained activation of XBP1 splicing leads to endothelial apoptosis and atherosclerosis development in response to disturbed flow. *Proceedings of the National Academy of Sciences*. 2009; 106:8326–8331.
