# Supplementary material for: Resveratrol-Induced Vascular Progenitor Differentiation towards Endothelial Lineage via MiR-21/Akt/β-Catenin Is Protective in Vessel Graft Models
Source: PLoS One. 2015 May 11;10(5):e0125122. doi: 10.1371/journal.pone.0125122 (PMC4427364; doi:10.1371/journal.pone.0125122)
Supplement: S1 Table — Primer sequences. (PDF) [file pone.0125122.s006.pdf]

Supplemental Table 1: Primer sequences

Gene Target	Sequence (5'→ 3')
β-actin	GGCTGTATTCCCCTCCATCG<br>CCAGTTGGTAACAATGCCATGT
CD31	CAAACAGAAACCCGTGGAGAT<br>ACCGTAATGGCTGTTGGCTTC
VE-cadherin	AAGAAACCGCTGATCGGCA<br>TCGGAAGAATTGGCCTCTGTC
eNOS	GGCTGGGTTTAGGGCTGTG<br>CTGAGGGTGTCGTAGGTGATG
SMA	TCCTGACGCTGAAGTATCCGAT<br>GGCCACACGAAGCTCGTTATAG
β-catenin	TGCAGCTTCTGGGTTCCGATGATA<br>AGATGGCAGGCTCAGTGATGTCTT
SM22	GATATGGCAGCAGTGCAGAG<br>AGTTGGCTGTCTGTGAAGTC
PTEN	TGGATTCGACTTAGACTTGACCT<br>GCGGTGTCATAATGTCTCTCAG
